# Investigations Regarding the Addition of ZnO and Li_2_O-TiO_2_ to Phosphate-Tellurite Glasses: Structural, Chemical, and Mechanical Properties

**DOI:** 10.3390/ma15051644

**Published:** 2022-02-22

**Authors:** Mihail Elisa, Stefan-Marian Iordache, Ana-Maria Iordache, Constantina Raluca Stefan, Ileana Cristina Vasiliu, Daniel Cristea, Doru Ursutiu, Cornel Samoila, Bogdan Alexandru Sava, Lucica Boroica, Marius Catalin Dinca, Ana Violeta Filip, Mihai Eftimie, Monica Enculescu

**Affiliations:** 1 Optospintronics Department, National Institute of R & D for Optoelectronics INOE 2000, 077125 Magurele, Romania; elisa@inoe.ro (M.E.); ana.iordache@inoe.ro (A.-M.I.); raluca.iordanescu@gmail.com (C.R.S.); icvasiliu@inoe.ro (I.C.V.); 2 Materials Science and Engineering Faculty, Transilvania University of Brasov, 500036 Brasov, Romania; csam@unitbv.ro; 3 Electrical Engineering and Computer Science Faculty, Transilvania University of Brasov, 500036 Brasov, Romania; udoru@unitbv.ro; 4 Academy of Romanian Scientists, 050044 Bucharest, Romania; 5 Technical Sciences Academy of Romania, 030167 Bucharest, Romania; 6 Laser Department, National Institute for Lasers, Plasma and Radiation Physics, 077125 Magurele, Romania; boroica_lucica@yahoo.com (L.B.); catalin69@live.com (M.C.D.); ana.filip@inflpr.ro (A.V.F.); 7 Faculty of Applied Chemistry and Materials Science, Politehnica University of Bucharest, 060042 Bucharest, Romania; mihai.eftimie@upb.ro; 8 Multifunctional Materials and Structures Laboratory, National Institute of Materials Physics, 077125 Magurele, Romania; mdatcu@infim.ro

**Keywords:** mechanical properties, phosphate glass, scanning electron microscopy, tellurite glass, thermal expansion coefficient

## Abstract

Phosphate and tellurite glasses can be used in optics, optoelectronics, magneto-optics, and nuclear and medical fields. Two series of phosphate-tellurite glasses, (50-*x*)ZnO-10Al_2_O_3_-40P_2_O_5_-*x*TeO_2_ and (40-*x*)Li_2_O-10Al_2_O_3_-5TiO_2_-45P_2_O_5_-*x*TeO_2_ (*x* = 5, 10), were synthesized by a non-conventional wet-route, and the mechanical properties as key performance measures for their application in optoelectronics were investigated. X-ray Diffraction (XRD) measurements revealed the vitreous nature of the investigated materials. Instrumented indentation tests allowed the calculation of hardness (*H*) and Young’s modulus (*E*) using the Oliver and Pharr model. The influence of increasing the TeO_2_ content, as well as the substitution of ZnO by Li_2_O-TiO_2_, on the variation of hardness, Young’s modulus, penetration depth (*PD*), and fracture toughness (*FT*) was evaluated in both series. As a general trend, there is a decrease in the hardness and Young’s modulus with increasing penetration depth. The addition of Li_2_O and TiO_2_ instead of ZnO leads to improved hardness and elastic modulus values. Regarding the *H*/*E* ratio, it was found that the samples with lower TeO_2_ content should be significantly more crack-resistant compared to the higher TeO_2_ content samples. The *H*^3^/*E*^2^ ratio, being lower than 0.01, revealed a poor resistance of these glasses to plastic deformation. At the same time, a decrease of the fracture toughness with increasing TeO_2_ content was noticed for each glass series. Based on dilatometry measurements, the thermal expansion coefficient as well as the characteristic temperatures of the glasses were measured. Field Emission Scanning Electron Microscopy-Energy Dispersive X-ray analysis (FESEM-EDX) revealed a uniform distribution of the elements in the bulk samples. The mechanical properties of these vitreous materials are important in relation to their application as magneto-optical Faraday rotators in laser cavities.

## 1. Introduction

Mechanical properties of phosphate glasses and glass ceramics were investigated in relation to their application as solid electrolytes in Na-ion batteries [1]. It was found that glass ceramics obtained by an appropriate heat treatment of the starting glass exhibit an increased microhardness and Young’s modulus compared to the vitreous material. Improved mechanical properties of Na_2_O-CaO-Al_2_O_3_-Ga_2_O_3_-P_2_O_5_ (NCAGP) glass with an Ag^+^ → Na^+^ ion exchange process were reported [2]. The ion exchanged NCAGP glass is a potential electrolyte material for Na-batteries. The addition of up to 6 mol. % B_2_O_3_ in the phosphate glass content results in an increase of the flexural strength and hardness of the glass as well as a small decrease of the fracture toughness [3]. The electrochemical–mechanical modelling of all-solid-state lithium-ion batteries, including glasses and glass ceramics, has been very recently presented as a promising candidate for next-generation energy storage and sensor applications [4,5,6,7].

Increased values of hardness, Young’s modulus and fracture toughness of Bi, Pb-doped borophosphate glasses were reported [8] in comparison with the un-doped phosphate glasses [9] and rare-earth-doped phosphate glasses [10], due to the mixed glass former role of P_2_O_5_ and B_2_O_3_ components, which contribute to structural strengthening. The deformation of phosphate glasses under different load (strain) rates depends on the evolution of four mechanisms, namely, elastic deformation, densification, shear flow and fracture [11]. For reduced indentation loads, the first two mechanisms are prevalent, whereas shear flow and fracture are predominant for high-applied loads.

Optical components based on vitreous phosphate materials, such as mirrors, telescopes, lenses, etc., require adequate mechanical strength due to their handling and mounting in optical and optoelectronic devices [12,13,14]. The mechanical resistance of the phosphate glasses was reported to be improved by noble metal precipitation in the vitreous network, after appropriate heat treatment [15].

Tellurite glasses were investigated for their applications in solid electrolyte batteries [16]. It was found that the embedding of AgI in the glass network reduces the rigidity and the mechanical strength of the vitreous material. Mechanical properties of tellurite glasses have also been investigated for their applications in optoelectronic devices [17,18,19,20]. Thus, incorporation of Ag^+^ ions in the structure of zinc tellurite glasses [17] and lead tellurite glasses [18] enhances the mechanical properties and strengthens the vitreous structure. The increase in the Er_2_O_3_ content in zinc tellurite glasses [19] increases the mechanical strength of these vitreous materials. Elastic properties of WO_3_-B_2_O_3_-MgO-TeO_2_ and Ag_2_O-V_2_O_5_-MoO_3_-TeO_2_ glasses were also presented in correlation with the mixed former effect of WO_3_, MoO_3_, TeO_2,_ and B_2_O_3_ compounds [20].

In this work, results concerning the mechanical properties of some aluminum phosphate-tellurite glass compositions containing ZnO and Li_2_O-TiO_2_, respectively, are presented. The hardness, Young’s modulus, and fracture toughness of these vitreous materials were investigated in correlation with their composition and structure [21]. The main goal of the paper is related to the study of the structural, mechanical, thermal, and morphological properties of phosphate-tellurite glasses, taking into consideration their potential application for magneto-optical Faraday rotators. 

## 2. Materials and Methods

ZnO and Li_2_O-TiO_2_-containing phosphate glasses studied in this work belong to the oxide systems (50-*x*)ZnO-10Al_2_O_3_-40P_2_O_5_-*x*TeO_2_ (*x* = 5, 10) (henceforth named as Series A glasses) and (40-*x*)Li_2_O-10Al_2_O_3_-5TiO_2_-45P_2_O_5_-*x*TeO_2_ (*x* = 5, 10) (henceforth named as Series B glasses), respectively. These vitreous materials were synthesized by a non-conventional wet-route of starting reagents that involves the incorporation of the starting analytical purity solid reagents (ZnO, Li_2_CO_3_, Al_2_O_3_, TiO_2_, TeO_2_) into an 85% H_3_PO_4_ aqueous solution followed by evaporation by heating at 150 °C, under constant mechanical stirring. The steps of the experimental procedure were: (1) homogenization-evaporation of the batch; (2) melt-quenching of the batch; (iii) glass annealing; and (iv) optical processing of glasses. All the chemical reagents were purchased from Sigma-Aldrich. The benefits of the wet non-conventional technique are reported in several works of the authors [21,22,23,24] that show the role of this method in increasing the chemical and optical homogeneity of the final glasses, taking into consideration the molecular level mixing of the solid reagents in H_3_PO_4_ aqueous solution. In Table 1, the preliminary heat treatment and melting–annealing parameters of the prepared glasses are shown.

The homogenization of the glass batches was performed by using an alumina stirrer (Morgan, Fairfield, NJ, USA) at an average rate of 200 rot/min in alumina crucibles (SEPADIN, Romania) in order to improve the optical homogeneity of the final materials by the reduction of the gaseous inclusions and embedded striae. The casting of the glass melt occurred in preheated graphite molds followed by subsequent annealing to remove the remnant stress from the final product.

The X-ray diffraction (XRD) measurements were performed at room temperature, in the 10° to 80° range, with a 0.05° step and 3 s integration time, using a Bruker D8 Advance device (Bruker, Billerica, MA, USA) (CuKα, λ = 1.54056 Å).

For the mechanical characterization stages, the glass samples were embedded in low contraction resin, followed by polishing up to a mirror-like finish.

The hardness (*H*), elastic modulus (*E*), and fracture toughness (*FT*) were assessed by instrumented indentation using an NHT^2^ indenter from CSM Instruments/Anton Paar (Peseux, Switzerland) equipped with a diamond Berkovich geometry three-sided pyramidal tip. Prior to the measurement stage, tip calibration was performed using a fused silica caliber to overcome the effect of tip rounding. Sets of 5 indentations per load, up to the desired value, were performed on each glass sample (10 mN, 50 mN, 100 mN, 200 mN, and 400 mN maximum loads) in different areas. The indentation protocol was: approach speed 2000 nm/min, 30 s loading stage, 3 s dwell time (to minimize the creep effect), and 10 s unloading stage. The loading–unloading curves were processed using the Oliver–Pharr method [25,26].

In order to determine the fracture toughness (*FT*) of brittle materials using indentation tests, several models have been advanced over the last few decades [27,28,29,30,31,32]. The *FT* parameter was calculated for each glass sample using the model proposed in [32,33], based on the *H* and *E* values determined from indentation tests, the lengths of the cracks from the corners of the imprints, and the applied maximum load. Thus, sets of 6 nanoindentation tests were performed on each glass sample in different areas, with a maximum load of 500 mN, while keeping the remaining parameters identical to the previous measurements. The length of the cracks was assessed by optical microscopy, and the results were averaged.

The thermal expansion coefficient was determined by the dilatometry method using a horizontal Netzsch-Gerätebau DIL 400 PC device (Germany). The temperature was increased by 3 °C/min. The characteristic temperatures (strain point, *T_S_*, glass transition temperature, *T_g_*, annealing point, *T_A_*, and softening point, *T_D_*), as well as the thermal expansion coefficient ($\alpha_{20}^{300}$) of the glass, were assessed by processing the results from the dilatometry measurements. The measurement errors for the thermal expansion coefficient were in the range ±0.8 × 10^−7^ K^−1^, provided by calibration with a SiO_2_ standard sample, according to ISO 7991.

The morphology and the elemental composition of the phosphate-tellurite glasses were evaluated using a Gemini 500 Carl Zeiss field emission scanning electron microscope (FESEM) (Carl Zeiss, Oberkochen, Germany) equipped with a Bruker QUANTAX 200 Energy Dispersive X-ray Spectrometer (EDX) (Brucker, Bremen, Germany), with an XFlash 6 silicon drift detector, energy resolution < 129 eV at Mn-Ka, and Peltier cooling. FESEM images at good resolution were acquired without covering the samples with metallic gold.

## 3. Results and Discussions

### 3.1. X-ray Diffraction 

**Figure 1:**
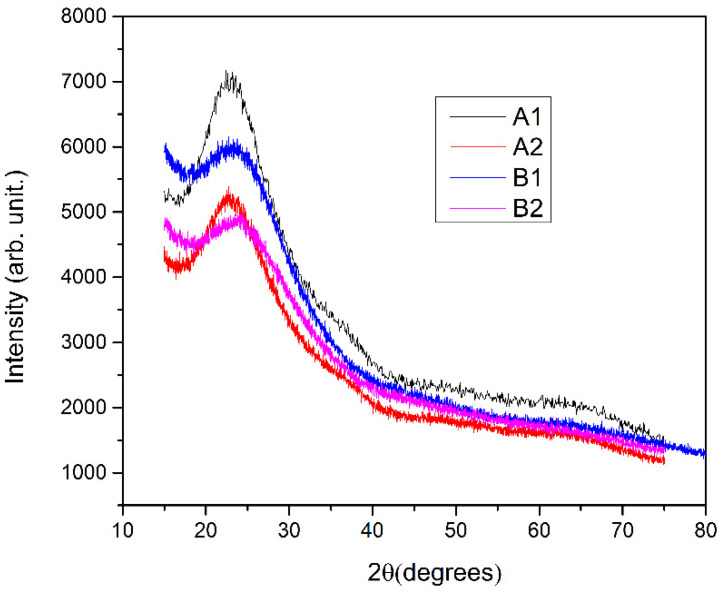
XRD patterns of A1, A2, B1, and B2 glasses.

The X-ray diffraction pattern of A1, A2, B1, and B2 glasses were collected at room temperature and are shown in Figure 1. The graphs corroborate the amorphous nature of the explored materials.

### 3.2. Hardness and Elastic Modulus

The penetration depth (*PD*), as a result of the indentation tests for the applied loads (10 mN, 50 mN, 100 mN, 200 mN, and 400 mN) on each glass, is influenced by the chemical composition of the investigated vitreous materials.

**Figure 2:**
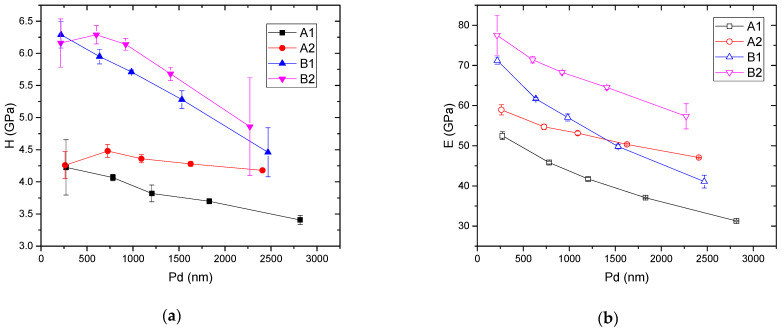
The variation of hardness (*H*) (**a**) and indentation elastic modulus (*E*) (**b**), as a function of penetration depth (*PD*).

Table 2 presents the average values for *H*, *E*, and the *H*/*E* and *H*^3^/*E*^2^ ratios, all as a function of the applied load and, consequently, as a function of *PD*. At the subsurface level, series A samples exhibit poorer mechanical behavior, compared to series B samples. This phenomenon would signify that the addition of Li_2_O and TiO_2_, instead of ZnO, leads to enhanced *H* and *E* values. This observation is better visualized in the graph from Figure 2, where one can observe the variation of *H* (a) and *E* (b), as function of the *PD*, for all the samples presented herein.

As a general trend, one can notice a decrease of *H* and *E* values with increasing penetration depth. In the case of ZnO-containing glasses (Series A), *H* and *E* have higher values when the content of TeO_2_ increases and ZnO content decreases. In the case of Li_2_O-TiO_2_-containing glasses (Series B), the increase in TeO_2_ content does not significantly influence the hardness; however, the difference in the variation of the elastic modulus is more obvious, with sample B2 exhibiting higher values for this parameter. The substitution of ZnO by Li_2_O-TiO_2_ for the same TeO_2_ content (sample B2 compared to sample A2) results in a significant increase in the mechanical parameter values. ZnO is generally used to improve the glass quality because it contributes to an increase of the chemical stability and durability [34,35,36] as well as high *E* values [37]. Our findings show that the addition of ZnO cannot be considered a foolproof solution to improve the mechanical characteristics of such glasses. It was shown elsewhere that gradual replacement of TeO_2_ with Li_2_O in the (70-*x*)TeO_2_ + 15B_2_O_3_ + 15P_2_O_5_ + *x*Li_2_O (*x* = 5, 10, 15, 20, 25, 30 mol. %) glass system leads to the decrease of the average crosslink density and rigidity of the prepared samples, which affects the mechanical properties (i.e., *H* and *E* values are decreased) [38]. In the case of the explored phosphate-tellurite glasses from this work, the *E* values of Li_2_O-TiO_2_-containing glasses are higher than those of ZnO-containing glasses for the same TeO_2_ content. As a conclusion, it can be stated that the increase in TeO_2_ content, as well as the replacement of ZnO by Li_2_O-TiO_2_, determines an increase in *H* and *E* values. This is in agreement with [39], where it was shown that the addition of TiO_2_ in (95-*x*)TeO_2_-5La_2_O_3_-*x*TiO_2_, (*x* = 0–20 mol. %) glasses caused an increase of *H* and *E* values. This is due to the formation of Ti-O-Ti bonds that caused an increase in stability and stiffness of the glasses. The same effect of TiO_2_ addition to the increasing of *E* values and glass durability was found for bioactive phosphate glasses reported in [40,41]. Some reference values of *H* and *E* for different phosphate glasses are presented elsewhere [9].

Apart from hardness, it is clear that Young’s modulus has a crucial role in the mechanical behavior of a material. The *H*/*E* ratio, called elastic strain to failure, can be a suitable parameter with the ability to predict, to a certain extent, the wear resistance of materials. Materials with high *H*/*E* ratio values generally exhibit better wear and failure resistance. This ratio can be used to predict whether the material will crack or not under load. Materials with high hardness and relatively lower values of Young’s modulus should exhibit improved fracture toughness. Materials with a high *H*/*E* ratio have a high plasticity index, meaning that their deformation under load is more likely to be elastic than plastic. Consequently, it is desirable to obtain a large *H*/*E* value, meaning sufficiently high hardness (to resist plastic deformation), but with a low elastic modulus. According to previous studies, materials with *H*/*E* ≥ 0.1 are considered to be resistant to cracking [41,42].

Furthermore, the *H*^3^/*E*^2^ ratio is a strong indicator of the resistance of a material to plastic deformation, a ranking parameter for prediction of shear banding under localized deformation. The *H*^3^/*E*^2^ ratio is not generally used to assess the wear and failure resistance of a material; however, it gives an estimation regarding its elasticity [43]. Materials with *H*^3^/*E*^2^ ≥ 0.1 are estimated to be resistant to plastic deformation. 

**Figure 3:**
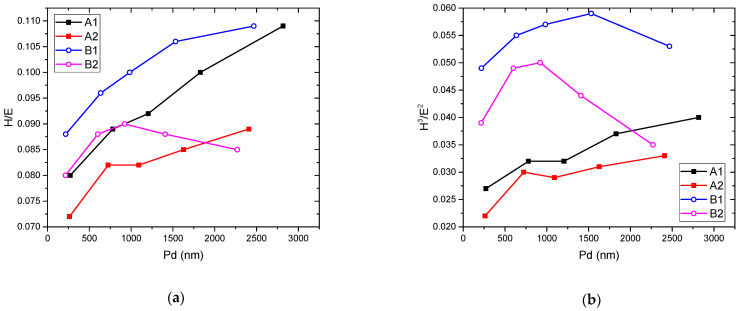
The variation of *H*/*E* (**a**) and *H*^3^/*E*^2^ (**b**) ratios, as a function of the penetration depth.

The variation of these two ratios, based on the *H* and *E* values obtained through instrumented indentation, as a function of penetration depth is presented in Figure 3. One can observe that if the previously mentioned predictions are accurate, the samples with lower TeO_2_ content should be significantly more resistant to cracking compared to the remaining samples.

As far as the *H*^3^/*E*^2^ ratio is concerned, considering that the maximum value exhibited by the samples presented herein is below 0.06, these glasses would exhibit poor resistance to plastic deformation.

**Figure 4:**
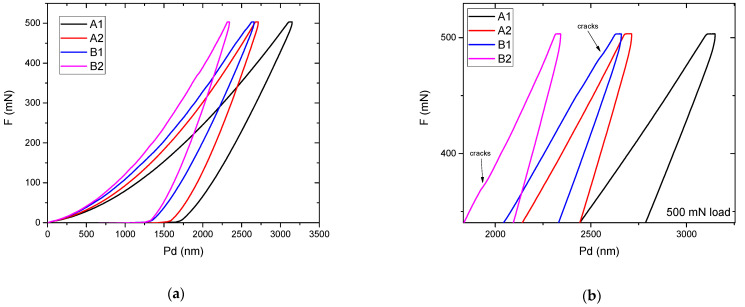
The loading—unloading curves obtained using 500 mN load (**a**); detail of the same curves, showing small deviations which signify the occurrence of cracks (**b**).

Figure 4 represents the loading–unloading curves obtained on these glasses, with an applied load of 500 mN. It is obvious from the lower penetration depth values for the same applied load that the samples from series B are harder, as shown in the previous paragraphs. Furthermore, a particular phenomenon can be inferred from the loading section of these curves, shown in Figure 4b (magnified). Sudden increases in the *PD* values, such as those observed for samples B1 and B2, can signify several events happening in the material: micro-cracks, phase transformations, dislocations, nucleation, and strain transfer across grain boundaries, all due to the applied load. Knowing that the materials in question are amorphous, these sudden increases in *PD* values can be accurately linked with the occurrence of cracks in the material. Consequently, the *FT* analysis, based on the occurrence of these cracks, will be presented in the next subsection.

### 3.3. Fracture Toughness

**Figure 5:**
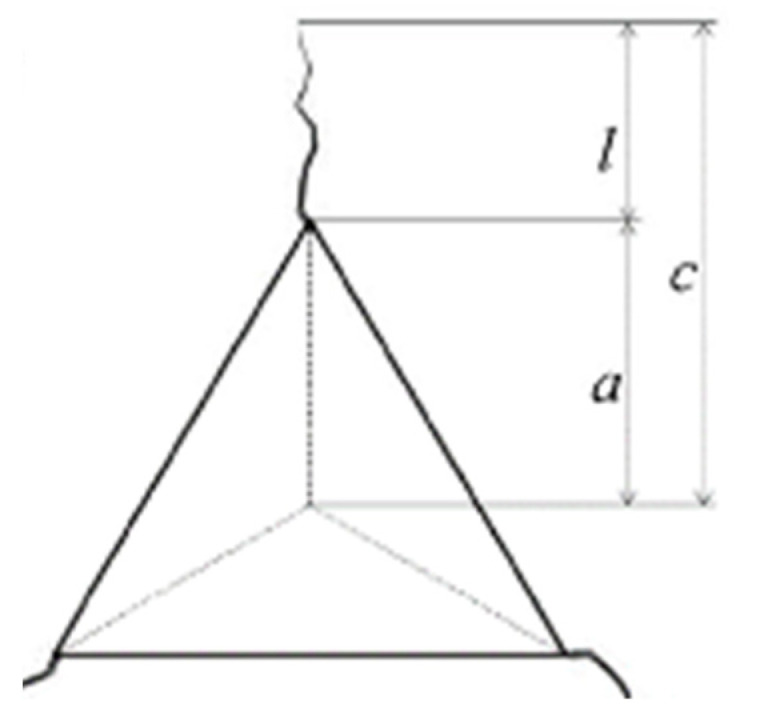
Indentation-induced crack geometry for Berkovich tips.

Figure 5 shows schematically the geometrical characteristics needed for calculating the *FT* values, based on Berkovich imprints obtained with 500 mN loads. The propagation of radial cracks from the corners of the imprint to the outside, in the bulk material, is of particular interest. Three parameters are important: (i) length *a*, measured from the center of contact to the end of the crack corner; (ii) the crack length *l*, measured from the corner of each imprint; and (iii) length *c*, which is the total length, *a* + *l*. The asymmetric shape of the Berkovich indenter type does not allow the cracks to join two corners of the imprint by passing through the center of the indentation. Therefore, the formation of radial cracks is more likely to occur. Six nanoindentation tests with an applied load of 500 mN were performed. The averaged values for *a*, *c*, and *l*, and the calculated *FT* parameter (*K_c_*), are presented in Table 3.

The crack parameters *a*, *l*, and *c*, were used to calculate the *FT* parameter Equation (1), taking into account the average values of *H*, *E*, *PD*, and applied load. 

Dukino and Swain [30] changed the method proposed in [31] with a correction factor, initially proposed in [32], to adjust the analysis of cracks induced by Berkovich tip geometry, as shown in Equation (1):

(1) $$K_{c}=1.073x_{v\;}{\left(a/l\right)}^{1/2}{\left(E/H\right)}^{2/3}\;\;\left(P/c^{3/2}\right)$$

where *x_v_* = 0.015 is an empirical calibration constant, *a*, *l*, and *c* are the crack length (m) parameters (see Figure 5), *E* is Young’s modulus (Pa), *H* is the hardness (Pa), and *P* is the applied load (N). It was noticed that the (*P*/*c*^3/2^) ratio is almost constant and dependent on the material properties [28]. From Table 3, in the case of ZnO-containing glasses, a decrease of *FT* parameter with increasing TeO_2_ content is noticed. The same trend is noticed in the case of Li_2_O-TiO_2_-containing glasses with increasing TeO_2_ content. A more evident decrease is observed when ZnO is replaced by Li_2_O-TiO_2_ for a constant TeO_2_ content. Some reference values related to the *FT* parameter for different phosphate glasses are presented elsewhere [9].

### 3.4. Dilatometry Measurements

The characteristic temperatures as well as the thermal expansion coefficient values for the glasses from the A and B series are presented in Table 4. The thermal expansion coefficient parameter was determined by the dilatometry method in the 20–300 °C range and it expresses the ratio between elongation and the initial length of the sample.

Taking into consideration the nominal molar formula of the synthesized glass samples, it is possible to state that the vitreous network is built by metaphosphate chains and crosslinked metaphosphate chains formed by P-O-P, P-O-Te and Te-O-Te bonds, revealing the vitreous network-forming role of P_2_O_5_ and TeO_2_ [21,44].

B series glasses exhibit thermal expansion coefficient values higher than those of the A series, which could be explained by the formation of a less rigid network, characterized by lower energy bonds values and, consequently, lower characteristic temperatures, strain point, *T_S_*, glass transition temperature, *T_g_*, annealing point, *T_A_*, and softening point, *T_D_* (see Table 4). The reason is the presence of Li_2_O and TiO_2_, whose role is to modify the vitreous network, breaking the metaphosphate chains and resulting in non-bridging oxygen atoms, O^−^ linked by Li^+^ and Ti^4+^ cations. The energy of the bonds, in this case, is lower than in the case of bridging oxygen atoms P-O-P, P-O-Te, and Te-O-Te, causing a decrease of the network stiffness and higher values of the hardness and elastic modulus as compared to the A glass series. This can explain a reduced remnant deformation, showing in evidence a more pronounced elastic behavior in relation to the A glass series (see Figure 4).

In the case of the A glass series, ZnO in a high amount takes the role of vitreous network former by creating Zn-O-P and Zn-O-Te bonds, consolidating the connections within the phosphate chains and the three-dimensional structures. This can clarify the reduced values of hardness and elastic modulus and higher thermal strength compared to the B glass series. Finally, the less rigid structure and the binding energies with lower values in the case of the B glass series could also elucidate the lower values of the crack resistance coefficients compared to those of the A glass series.

### 3.5. FESEM-EDX Measurements

The samples were analyzed in several zones in order to acquire elemental maps showing in evidence the nano-clustering of diatomic tellurium molecules [45]. The elemental mapping did not reveal these nanoaggregates, tellurium atoms being relatively evenly distributed in the bulk samples.

**Figure 6:**
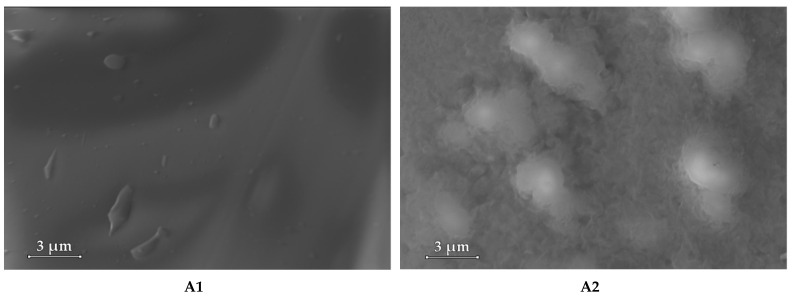

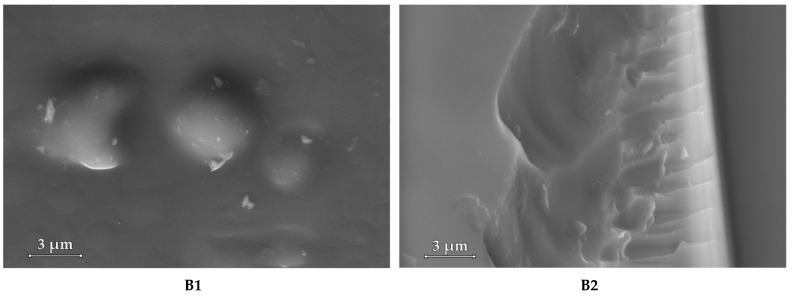
Surface morphology of the A1, A2, B1, and B2 glass samples.

**Figure 7:**
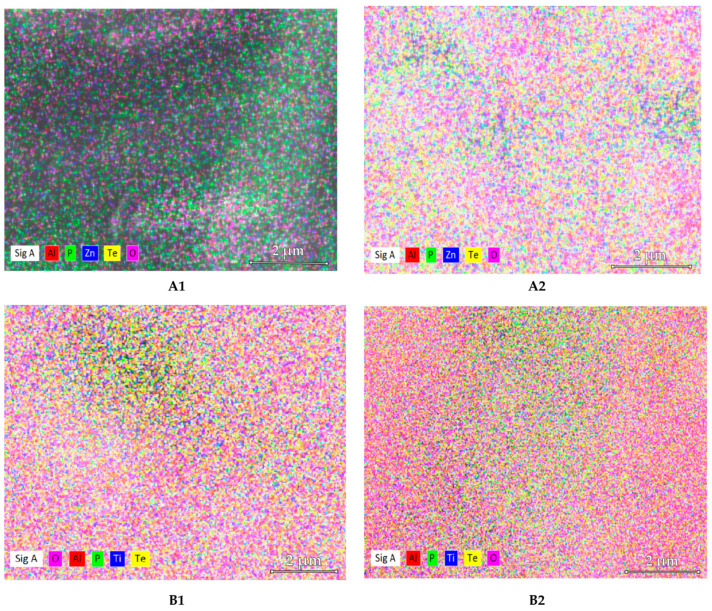
Elemental maps of the A1, A2, B1, and B2 glass samples.

Figure 6 exhibits the morphology of the glass samples, obtained by FESEM, while Figure 7 shows the elemental maps of the A1, A2, B1, and B2 glass samples.

Figure 7 reveals the distribution of the elements (Zn, Al, P, O, Te, and Ti) regarding their dispersion and concentration in the analyzed samples.

Table 5 displays the comparison between the wt. % nominal elemental composition and the composition obtained from EDS for the A1, A2, B1, and B2 glasses. 

Lithium could not be detected by EDX due to the low atomic weight, the emission of light elements not being analyzed using this technique. Thus, the EDX elemental composition of B1 and B2 samples presented in Table 5 was calculated considering the lithium percentage in the prepared samples equal to the nominal percentage, and the elemental composition provided by the EDX algorithm for the other elements was normalized by taking into account the lithium percentage.

Although the FESEM images evidenced the presence of small formations in all the evaluated glasses, when analyzing the dispersion and the concentration of the elements in the samples by using EDX elemental mapping, a uniform distribution of the elements was observed. From Table 5, it is observed that, mainly in the case of A2, B1, and B2 glasses, close values of the nominal elemental composition and EDX elemental composition were found.

## 4. Conclusions

In the case of ZnO-containing glasses, corresponding to the A glass series ((50-*x*)ZnO-10Al_2_O_3_-40P_2_O_5_-*x*TeO_2_ (*x* = 5, 10)), the hardness increases with TeO_2_ content, whereas in the case of Li_2_O-TiO_2_-containing glasses, corresponding to the B glass series ((40-*x*)Li_2_O-10Al_2_O_3_-5TiO_2_-45P_2_O_5_-*x*TeO_2_ (*x* = 5, 10)), the increase in TeO_2_ content does not considerably influence the hardness of the glasses. The hardness of Li_2_O-TiO_2_-containing glasses is higher than that of ZnO-containing glasses for the same TeO_2_ content. Young’s modulus, in the case of ZnO-containing glasses, increases with TeO_2_ content, the same trend being remarked in the case of Li_2_O-TiO_2_-containing glasses. Young’s modulus of TiO_2_-containing glasses is higher than that of ZnO-containing glasses for the same TeO_2_ content. It can be stated that the increase in TeO_2_ content, as well as the replacement of ZnO by Li_2_O-TiO_2_, determines an increase of the hardness and Young’s modulus.

In the case of ZnO-containing glasses, the increase of TeO_2_ content results in a decrease of fracture toughness (*FT* parameter). The same trend is noticed in the case of Li_2_O-TiO_2_-containing glasses with increasing TeO_2_ content. A more evident decrease of *FT* is observed when ZnO is replaced by Li_2_O-TiO_2_ for a constant TeO_2_ content.

The thermal expansion coefficient values in the case of the B glass series are higher than those of the A glass series due to the less rigid network. Consequently, the characteristic temperatures in the case of the B glass series are lower than those of the A glass series. The presence of different vitreous network modifiers could be the source of the higher hardness and elastic modulus values in the case of the B glass series as compared to those of the A glass series.

FESEM images and EDX elemental maps evidenced a relatively uniform distribution of the constituent elements in the structure of the bulk glasses, ruling out the variation in mechanical behavior due to potential inhomogeneities in the samples.

The correlation between the presence of different vitreous network modifiers and the mechanical properties of the investigated glasses is essential for their application as magneto-optical Faraday rotators.

Future work will be developed related to research of phosphate-tellurite glasses containing post transition and heavy metal ions with magnetic and magneto-optical properties for Faraday rotators. 

## Figures and Tables

**Table 1:** Preliminary heat treatment and melting–annealing parameters of the prepared glasses (*T_g_* is the vitreous transition temperature determined from the thermal expansion curves measured in the 20–500 °C range, not presented in this work).

Glass Code	Molar Composition	Preliminary Heat Treatment Parameters	Melting and Refining Parameters	Annealing Parameters	Glass Aspect
A1	45ZnO 10Al_2_O_3_ 40P_2_O_5_ 5TeO_2_	Homogenization & evaporation at 200 °C, for 1 h, followed by heat treatment at 700 °C, for 7 h	Melting at 1100 °C, for 30 min, followed by refining at 1125 °C, for 30 min	Plateau at 390 °C, for 30 min, followed by gradually quenching (T_g_ = 404 °C)	Transparent mauve reddish color
A2	40ZnO 10Al_2_O_3_ 40P_2_O_5_ 10TeO_2_	Homogenization & evaporation at 200 °C, for 0.5 h, followed by heat treatment at 700 °C, for 2.5 h	Melting at 1100 °C, for 30 min, followed by refining at 1125 °C, for 30 min	Plateau at 400 °C, for 30 min, followed by gradually quenching (T_g_ = 429 °C)	Transparent dark reddish color
B1	35Li_2_O 10Al_2_O_3_ 5TiO_2_ 45P_2_O_5_ 5TeO_2_	Homogenization & evaporation at 200 °C, for 25 min, followed by heat treatment at 700 °C, for 2.5 h	Melting at 1225 °C, 30 min, followed by refining at 1250 °C, for 30 min	Plateau at 420 °C, for 0.5 h, followed by gradually quenching (T_g_ = 419 °C)	Transparent dark reddish—brown color
B2	30Li_2_O 10Al_2_O_3_ 5TiO_2_ 45P_2_O_5_ 10TeO_2_	Homogenization & evaporation at 200 °C, for 60 min, followed by heat treatment at 240 °C, for 2 h, and 700 °C, for 3 h	Melting at 1200 °C, for 30 min, followed by refining at 1225 °C, for 30 min	Plateau at 400 °C, for 30 min, followed by gradually quenching (T_g_ = 427 °C)	Transparent dark brown color

**Table 2:** Average values of *H*, *E*, *H*/*E*, and *H*^3^/*E*^2^ ratios.

Glass Code	A1				A2				B1				B2			
Load	*H*	*E*	*H*/*E*	*H*^3^/*E*^2^	*H*	*E*	*H*/*E*	*H*^3^/*E*^2^	*H*	*E*	*H*/*E*	*H*^3^/*E*^2^	*H*	*E*	*H*/*E*	*H*^3^/*E*^2^
10 mN	4.22	52.49	0.080	0.027	4.25	58.94	0.072	0.022	6.28	71.19	0.088	0.049	6.16	77.51	0.080	0.039
50 mN	4.06	45.81	0.089	0.032	4.47	54.70	0.082	0.030	5.95	61.67	0.096	0.055	6.28	71.41	0.088	0.049
100 mN	3.82	41.71	0.092	0.032	4.35	53.14	0.082	0.029	5.70	57.02	0.100	0.057	6.14	68.26	0.090	0.050
200 mN	3.69	37.05	0.100	0.037	4.28	50.32	0.085	0.031	5.28	49.80	0.106	0.059	5.68	64.53	0.088	0.044
400 mN	3.40	31.26	0.109	0.040	4.18	47.05	0.089	0.033	4.46	41.07	0.109	0.053	4.86	57.31	0.085	0.035
500 mN	3.45	29.97	0.115	0.046	4.15	45.65	0.091	0.034	4.96	40.82	0.122	0.073	5.66	59.49	0.095	0.051

**Table 3:** Crack parameters (*a*, *l* and *c*) and *K_c_*.

Glass Code	*a* (m) × 10^−8^	*l* (m) × 10^−8^	*c* (m) × 10^−8^	*K_c_* (MPa m^1/2^)
A1	1000	693	1693	0.594
A2	1018	840	1858	0.554
B1	894	920	1814	0.424
B2	879	1055	1934	0.420

**Table 4:** Characteristic temperatures and thermal expansion coefficient for A and B glass series.

Property/Glass Code	A1	A2	B1	B2
Strain point, T_S_	403	403	391	394
Glass transition temperature, T_g_	426	429	419	427
Annealing point, T_A_	441	437	426	436
Softening point, T_D_	448	453	439	446
$\mathrm{Thermal}\;\mathrm{expansion}\;\mathrm{coefficient},\;\propto_{20}^{300}$ × 10^−6^ (K^−1^)	6.86	6.56	11.67	11.95

**Table 5:** Nominal elemental composition and EDX elemental composition of A1, A2, B1, and B2 glasses.

Glass Sample	Element	Nominal Elemental Composition (wt. %)	EDX Elemental Composition (wt. %)
A1	Al	4.85	4.27
	P	22.25	14.05
	Zn	26.24	5.57
	Te	5.74	3.26
	O	40.92	72.85
Total		100.00	100.00
A2	O	40.21	49.82
	Al	4.68	3.53
	P	21.49	22.41
	Te	11.09	6.8
	Zn	22.53	17.44
Total		100.00	100.00
B1	Li	5.07	5.07
	O	51.35	56.32
	Al	5.59	4.46
	P	28.88	26.39
	Ti	2.48	2.89
	Te	6.63	4.87
Total		100.00	100.00
B2	Li	4.07	4.07
	O	48.88	46.33
	Al	5.24	6.89
	P	27.06	30.64
	Ti	2.33	2.39
	Te	12.42	9.68
Total		100.00	100.00

## Data Availability

Not applicable.
